# A rapid method for detection of five known mutations associated with aminoglycoside-induced deafness

**DOI:** 10.1186/1471-2350-10-2

**Published:** 2009-01-13

**Authors:** Soraya Bardien, Hannique Human, Tashneem Harris, Gwynneth Hefke, Rene Veikondis, H Simon Schaaf, Lize van der Merwe, John H Greinwald, Johan Fagan, Greetje de Jong

**Affiliations:** 1 Division of Molecular Biology and Human Genetics, Stellenbosch University, Cape Town, South Africa; 2 Division of Otolaryngology, Groote Schuur Hospital and University of Cape Town, Cape Town, South Africa; 3 Central Analytical Facility, Stellenbosch University, Cape Town, South Africa; 4 Department of Paediatrics and Child Health, Faculty of Health Sciences, Stellenbosch University, Cape Town, South Africa; 5 Tygerberg Children's Hospital, Cape Town, South Africa; 6 Biostatistics Unit, Medical Research Council of South Africa, Cape Town, South Africa; 7 Department of Otolaryngology-Head and Neck Surgery, Hearing and Deafness Center, University of Cincinnati College of Medicine, Cincinnati, USA; 8 Department of Pediatric Otolaryngology-Head and Neck Surgery, Cincinnati Children's Hospital Medical Center, Cincinnati, USA

## Abstract

**Background:**

South Africa has one of the highest incidences of multidrug-resistant tuberculosis (MDR-TB) in the world. Concomitantly, aminoglycosides are commonly used in this country as a treatment against MDR-TB. To date, at least five mutations are known to confer susceptibility to aminoglycoside-induced hearing loss. The aim of the present study was to develop a rapid screening method to determine whether these mutations are present in the South African population.

**Methods:**

A multiplex method using the SNaPshot technique was used to screen for five mutations in the *MT-RNR1 *gene: A1555G, C1494T, T1095C, 961delT+C(n) and A827G. A total of 204 South African control samples, comprising 98 Mixed ancestry and 106 Black individuals were screened for the presence of the five mutations.

**Results:**

A robust, cost-effective method was developed that detected the presence of all five sequence variants simultaneously. In this pilot study, the A1555G mutation was identified at a frequency of 0.9% in the Black control samples. The 961delT+C(n) variant was present in 6.6% of the Black controls and 2% of the Mixed ancestry controls. The T1095C, C1494T and A827G variants were not identified in any of the study participants.

**Conclusion:**

The frequency of 0.9% for the A1555G mutation in the Black population in South Africa is of concern given the high incidence of MDR-TB in this particular ethnic group. Future larger studies are warranted to determine the true frequencies of the aminoglycoside deafness mutations in the general South African population. The high frequencies of the 961delT+C(n) variant observed in the controls suggest that this change is a common non-pathogenic polymorphism. This genetic method facilitates the identification of individuals at high risk of developing hearing loss prior to the start of aminoglycoside therapy. This is important in a low-resource country like South Africa where, despite their adverse side-effects, aminoglycosides will continue to be used routinely and are accompanied with very limited or no audiological monitoring.

## Background

South Africa is rated 4th among the 22 high-burden tuberculosis (TB) countries by the World Health Organisation (WHO) [1]. A recent study showed that eight of the nine South African provinces (no data was available for the 9^th ^province) have an estimated incidence of greater than three per 100,000 of the population for multidrug-resistant tuberculosis (MDR-TB) [2]. In the Western Cape Province, the estimated incidence for MDR-TB is 8.39 per 100,000. Aminoglycoside antibiotics such as kanamycin and amikacin are part of the WHO's recommended treatment regimen for MDR-TB and streptomycin is used for re-treatment TB cases [3]. In South Africa, aminoglycosides are routinely used to treat severe Gram-negative bacterial infections but their usage is on the increase because of the high incidence of re-treatment TB and MDR-TB cases. These drugs have well-documented adverse reactions such as ototoxicity and nephrotoxicity. The nephrotoxicity is usually reversible but the ototoxicity, which is most likely due to damage to the sensory hair cells and the stria vascularis in the cochlea, is permanent [4].

In China, where there is also widespread use of aminoglycosides, the incidence of ototoxicity has been well documented. In a district of Shanghai, the cause of hearing loss could be traced to aminoglycoside usage in approximately 22% of all deaf mute individuals [5]. Although some of the earliest reports on this condition came from South Africa [6,7], no data on the incidence of aminoglycoside-induced deafness has ever been documented for this country.

To date, at least five different homoplasmic mutations in the mitochondrial gene encoding 12S rRNA (*MT-RNR1*) have been found to predispose individuals to irreversible hearing loss if they are treated with aminoglycoside antibiotics. These include A1555G [8], 961delT+C(n) [9], T1095C [10], C1494T [11] and A827G [12]. The A1555G mutation, which was the first one to be described, is the most common variant and has been reported in diverse populations worldwide [13]. Individuals harbouring A1555G can develop hearing loss even in the absence of aminoglycoside exposure [14]. Various methods have been used to screen for these mutations including allele-specific polymerase chain reaction (PCR) [15], DNA sequencing [16], PCR-restriction fragment length polymorphism (PCR-RFLP) analysis [14,16] and allele-specific oligonucleotide hybridisation (ASO) [17]. All these methods are limiting in that they can detect only one mutation at a time and are not amenable to multiplexing.

In the present study we aimed to develop a cost-effective multiplex genetic screening method in order to determine whether these five sequence variants are present in the South African population. Given that in South Africa aminoglycosides are commonly used, and that their usage will be on the increase in the near future to combat the rising MDR-TB epidemic it is important to determine the prevalence of these mutations. A method is needed to identify individuals or particular South African ethnic groups that are at increased risk of developing hearing loss prior to them starting aminoglycoside therapy.

## Methods

### Study participants

The study was approved by the Committee for Human Research at Stellenbosch University, South Africa (Protocol number: N05/09/165). Following informed written consent, blood samples from 204 controls from the Western Cape Province, consisting of 98 Mixed ancestry individuals and 106 Black individuals, were collected for genetic analysis. Only these two ethnic groups were investigated in the present study since the highest incidence of MDR-TB in this country occurs in these two sub-populations. South African Mixed ancestry represents predominantly an admixture of indigenous African populations, European immigrants from Western Europe and indentured labourers from Madagascar, the Malaysian archipelago and India [18].

### SNaPshot Analysis

Genomic DNA was extracted from blood samples collected from the study participants using established methods. PCR primers spanning the *MT-RNR1 *gene were designed using Primer3 software [19].

The SNaPshot technique (Applied Biosystems, Foster City, USA) is a method used specifically to genotype single nucleotide polymorphisms (SNPs). It involves PCR amplification of a region of interest, purification of the product and annealing of a SNaPshot primer that ends one nucleotide 5' of a known SNP. A single base extension reaction is then performed in the presence of the four fluorescently-labelled dideoxynucleotide triphosphates (ddNTPs). Upon excitation with a laser, the different fluorescent dyes emit a colour which is specific for each ddNTP i.e. green for A, blue for G, black for C and red for T. Multiple SNPs (up to 12) can be interrogated in a single SNaPshot reaction with the SNaPshot primers for the different SNPs varying in length. Therefore, following electrophoresis, each SNP's genotype is determined by both the position (size) of the peak as well as by the colour of the emitted fluorescence. In the case of homoplasmic mutations (such at the ones described in the present study), each variant's genotype is represented by a single peak e.g. for C1494T either a black peak or a red peak is present, for the C or the T allele, respectively. Initially, each mutation was analysed separately to establish the intervals for the observed peak locations (bins) for each allele. Thereafter, the five variants were multiplexed and analysed in a 'single-tube single-capillary' format. The length and orientation of the five SNaPshot primers underwent a series of redesigning and optimisation experiments until adequate separation of all the alleles representing the five different loci was achieved. The GeneMapper software (version 3.7; Applied Biosystems) was used to set up the bins for each allele for the mutations investigated and was also used for automated allele scoring and quality checking. Small non-specific peaks that were either below a certain threshold peak height or that did not fall into the specific bins were disregarded by the GeneMapper software and not genotyped.

The PCR and SNaPshot primers designed for the study are provided in Table 1. PCR reactions were performed in 50 μl reactions on a 2720 Thermal Cycler (Applied Biosystems, USA). The PCR cycling conditions comprised of an initial denaturation step of 94°C for 5 min, 30 cycles of denaturation at 94°C for 30 sec, annealing at 55°C for 30 sec, extension at 72°C for 45 sec, and a final extension step of 72°C for 7 min. Following PCR, 3 μl of the PCR product was incubated with 1 unit of shrimp alkaline phosphatase (SAP; Promega) and 0.2 units of *Exo I *(AEC Amersham) for 60 min at 37°C, followed by 30 min at 75°C for enzyme inactivation.

**Table 1:** Primers used for PCR amplification and SNaPshot extension reactions.

		**Primer sequences (5'-3')**	
**PCR primers**		For: caa cca aac ccc aaa gac ac	
		Rev: gct cag agc ggt caa gtt aag	
			
	**Variant**	**Primer sequences (5'-3')**	**Orientation**

**SNaPshot primers**			
	A1555G	ttg gca ttt ata tag agg ag	forward
	C1494T	cgt aca cac cgc ccg tca c	forward
	T1095C	ctg gga tta gat acc cca cta tgc t	forward
	961delT+C(n)	aca ggt gag ttt tag ctt tat tgg gg	reverse
	A827G	gct tag tta aac ttt cgt ttg ttg cta aag g	reverse

The SNaPshot extension reactions were carried out in a final volume of 10 μl containing 3 μl of purified PCR product, 3 μl of SNaPshot Ready Reaction Mix (ABI Prism SNaPshot Multiplex Kit) and 1.67 μl of a mixture of each of the five SNaPshot primers (each at 1.8 μM, except the primer for A1555G which was 2.9 μM). The cycling conditions were 27 cycles of 96°C for 10 sec, 50°C for 5 sec and 60°C for 30 sec. After primer extension, the unincorporated fluorescently labelled ddNTPs were removed by adding 1 unit of SAP and incubating for 60 min at 37°C followed by 30 min at 75°C for enzyme deactivation. One μl of each SNaPshot reaction, 0.5 μl Liz 120 size standard (Applied Biosystems) and 9 μl of HiDi formamide was loaded on an ABI 3130 × l Genetic Analyzer (Applied Biosystems) and electrophoresed using POP 7 polymer. All samples were genotyped using the GeneMapper software. Direct sequencing of selected samples was performed using the BigDye Terminator Sequence Ready Reaction kit version 3.1 (Applied Biosystems) and run on a 3130 × l Genetic Analyzer. The initial analysis was performed using Sequencing Analysis software (version 5.3.1) and BioEdit software (version 7.0.1) was used for interpretation of the sequencing electropherograms [20].

### Sequence alignments

To investigate the sequence conservation of *MT-RNR1*, multiple sequence alignments were performed using the Clustal W program (version 1.83). Sequences from the following six species were obtained from NCBI: human (NC_001807), chimp (NC_001643), gorilla (NC_001645), mouse (NC_005089), rat (NC_001665), dog (NC_002008) and cow (NC_006853).

## Results

**Figure 1:**
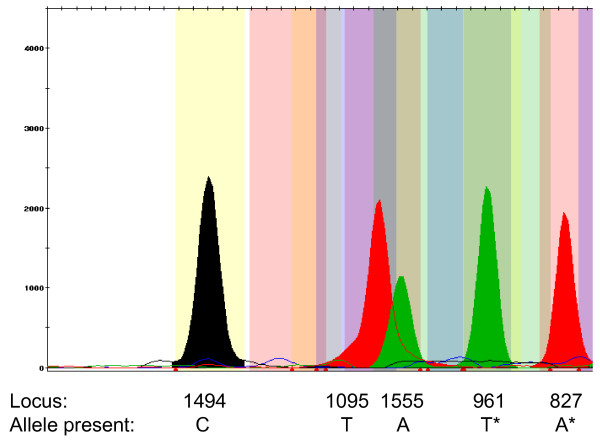
**SNaPshot analysis of the five mutations [C1494T, T1095C, A1555G, 961delT+C(n) and A827G]**. Peaks are shown that represent wild-type alleles, for one individual, at all five loci. The smaller non-specific peaks in the figure are all either below a certain threshold peak height value or do not fall into specific bins and are therefore not genotyped by the GeneMapper software. * The SNaPshot extension primers for 961 delT+C(n) and A827G are in the reverse orientation. In the SNaPshot figure, the genotypes are represented as follows: For C1494T, either a black peak (representing the C allele) or a red peak (T allele) is present, in their respective bin positions. For T1095C, either a red peak (T allele) or a black peak (C allele) is present, in their respective bin positions. For A1555G, either a green peak (A allele) or a blue peak (G allele) is present, in their respective bin positions. For 961delT+C(n) (as the primer anneals to the reverse strand), either a green peak (A allele; T on forward strand) or a blue peak (G allele; C on forward strand) is present, in their respective bin positions. For A827G (as the primer anneals to the reverse strand), either a red peak (T allele; A on forward strand) or a black peak (C allele; G on forward strand) is present, in their respective bin positions.

**Figure 2:**
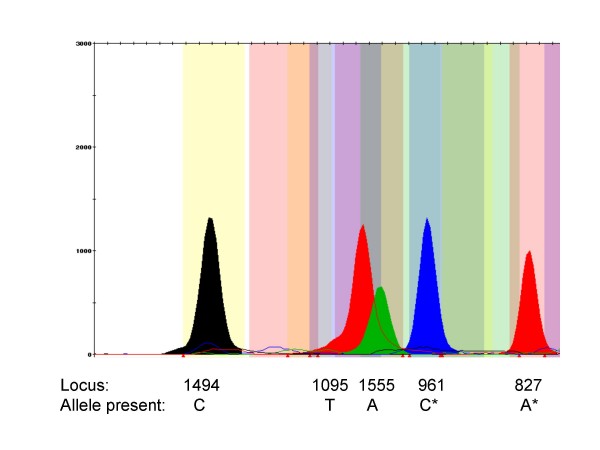
**SNaPshot analysis of an individual who harbours wild-type alleles at four of the loci and the mutant allele (delT) for 961delT+C(n)**. The five peaks each represent an allele as described in detail in the legend to Figure 1. * The SNaPshot extension primers for 961 delT+C(n) and A827G are in the reverse orientation.

A method for the simultaneous detection of five of the known aminoglycoside-induced deafness mutations has been developed in this study (Figures 1 and 2). PCR primers were designed that amplified the entire *MT-RNR1 *gene in a 1,124 bp PCR fragment for SNaPshot analysis. Using ABI GeneMapper software, bins were assigned at each of the five loci which facilitated high-throughput allele scoring and quality checking. At least one positive control for the mutant allele at each of the five loci was included in the analysis and was correctly genotyped in all runs. Furthermore, direct sequencing was performed on selected samples to verify the SNaPshot results and there was a 100% concordance.

**Figure 3:**
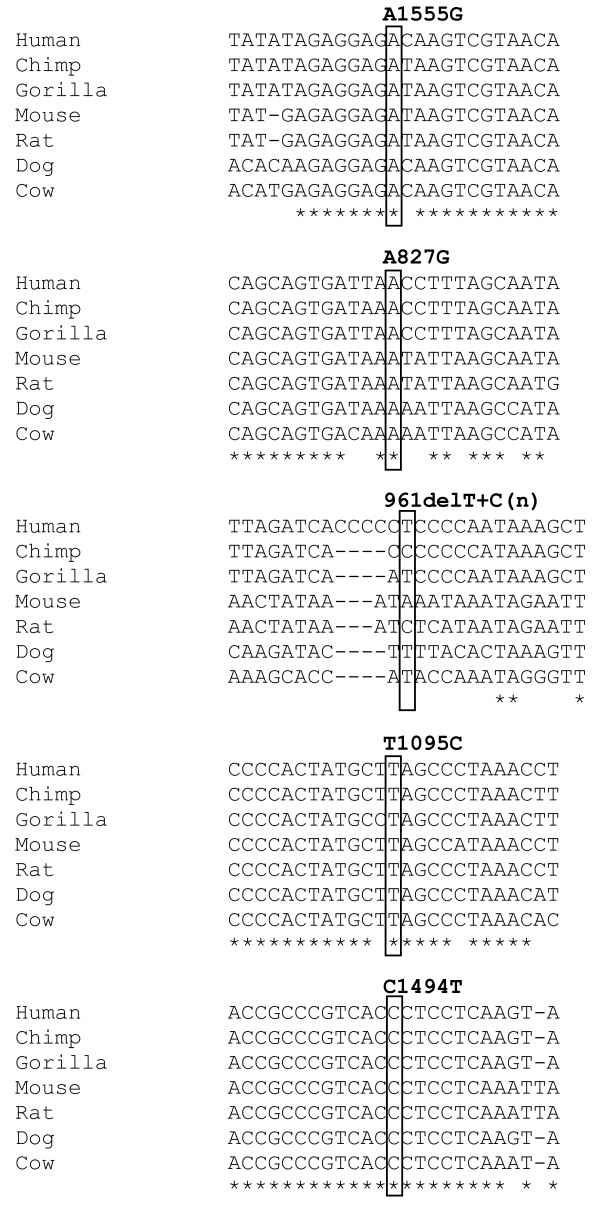
**Sequence alignments of the *MT-RNR1 *gene across diverse species indicating that the A1555, A827, T1095 and C1494 alleles are all evolutionarily conserved but that the T allele at 961 is not**.

The A1555G mutation was identified in one of the 106 Black control samples and in none of the Mixed ancestry controls (Table 2). This represents an observed frequency of 0.9% (95% CI 0.17% to 5.15%) for the A1555G mutation in the Black population in South Africa which is of concern given the high incidence of MDR-TB in this particular ethnic group. The A1555G mutation is a well-established aminoglycoside-induced deafness associated mutation and the A1555 allele is evolutionarily conserved across diverse species and is embedded in a conserved region of the gene (Figure 3).

**Table 2:** Frequency of the five aminoglycoside-induced deafness mutations in two South African populations.

	**Variants in *MT-RNR1***				
**Study participants**	**C1494T**	**T1095C**	**A1555G**	**961delT+C(n)**	**A827G**
Black controls n = 106	0	0	1 (0.9%)	7 (6.6%)	0
Mixed ancestry controls n = 98	0	0	0	2 (2.0%)	0

**TOTAL**	0	0	1	9	0

**Figure 4:**
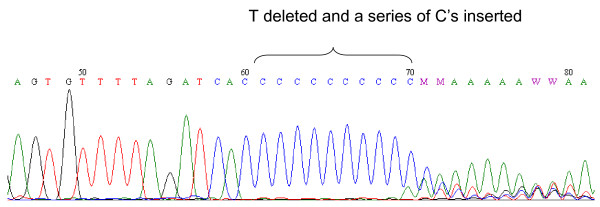
**Representative sequencing results confirming the presence of the 961delT+C(n) variant as detected by SNaPshot analysis**.

The 961delT+C(n) variant was identified at a relatively high frequency in this South African group of study participants. This variant was unambiguously detected using the SNaPshot technique. All 961delT+C(n)-positive samples were verified with sequencing to not be the other possible alleles at this locus including the 961insC or T961G variants (Figure 4). The 961delT+C(n) variant was observed in 6.6% of the Black controls and 2% of the Mixed ancestry controls (Table 2). These results indicate that the 961delT+C(n) variant may possibly be a common non-pathogenic polymorphism in the *MT-RNR1 *gene. This observation is corroborated by the fact that the T961 allele and its flanking residues are not evolutionarily conserved (Figure 3). The T1095C, C1494T and A827G variants were not identified in any of the study participants.

The method described in the present study was specifically designed and optimised to detect homoplasmic mutations, since, in the majority of published reports, the five mutations [A1555G, C1494T, T1095C, 961delT+C(n) and A827G] are present in the homoplasmic state. The SNaPshot method has, however, previously been shown to be a sensitive and reproducible method for the detection of heteroplasmic mutations in different tissues [21] but since no heteroplasmic mutations were detected in the present study, and as no heteroplasmic positive controls were available for testing, we cannot at this stage, comment on how well suited our method is for the detection of heteroplasmic mutations.

## Discussion

We have successfully developed a rapid, robust and cost-effective method for the screening of five of the known aminoglycoside-induced deafness mutations. The SNaPshot technique is particularly well-suited for the screening of mitochondrial DNA variants for the following reasons: i) the short intron-less mitochondrial genes means that the entire gene can be PCR-amplified and screened in a single PCR fragment and ii) homoplasmic mutations (such as the ones investigated in the present study) are represented as a single peak at each locus which simplifies the analysis. This method is also less labour-intensive and less prone to false positives compared to the other methods reported to date. In one study using PCR-RFLP and the enzyme, *Alw26I *to screen for A1555G, a false positive was detected: DNA sequencing revealed that the patient had in fact a T1556C variant, which also disrupts the *Alw26I *site [22]. In another study using ASO, the authors reported that this method was not effective for detection of the 961delT+C(n) variant [17]. In a low-resource country like South Africa, a typical alternative method used for SNP genotyping would be PCR-RFLP. An estimated cost comparison between the two methods (including DNA extraction) is ~$16 per sample for the SNaPshot method versus~$30 per sample for the PCR-RFLP method (using an exchange rate of $1 = 10.39 ZAR).

This is the first report on the frequency of aminoglycoside-induced deafness mutations in South African sub-populations. In the present pilot study, the A1555G mutation was present at a frequency of 0.9% in the South African Black control group. Further studies on larger numbers of study participants are needed to determine the true frequency of A1555G in this particular ethnic group but if the frequency is shown to be ≥ 1% this has important implications given the high incidence of TB and MDR-TB in this group. The A1555G has been found to be very uncommon in the general populations worldwide. It was found in 0.48% (1/206) [15], 0.09% (1/1,161) [17] and 0% (0/1,042) [23] of the general New Zealand, American and Argentinean populations, respectively.

The 961delT+Cn variant was detected at high frequencies in the study participants: 6.6% of the Black controls and 2% of the Mixed ancestry controls. Other studies have found this variant at a high frequency of controls and they also questioned the pathogenicity of this variant [17,24]. Furthermore, all the variants with the notable exception of 961delT+C(n) are evolutionarily conserved from man to cow which indicates their functional significance and that 961delT+Cn is likely to be a non-functional polymorphism. In a recent study it was shown that both A1555G and C1494T decrease the accuracy of translation in the mitochondrion and rendered the ribosomal decoding site hyper-susceptible to aminoglycosides [25]. Similar studies on 961delT+C(n), A827G and T1095C are needed to prove the pathogenicity of these variants and their possible effects on the functioning of the mitochondria. The T1095C, C1494T and A827G were not found in any of the study participants in the present study.

Future studies are warranted to determine the true frequencies of the various aminoglycoside deafness mutations in the general South African population and also whether certain aminoglycosides are more ototoxic than others. Despite their adverse effects, aminoglycosides are currently and in the future will continue to be used in developing countries like South Africa for re-treatment TB and MDR-TB where cost considerations are a major factor. South Africa is therefore an ideal setting to study this particular problem. In MDR-TB infections aminoglycoside administration is unavoidable and often has to be continued even though the patient experiences hearing loss. However, possible strategies to minimise the progression of cochlear and vestibular damage are: to identify high risk individuals who are mutation-positive and in these individuals to reduce the therapy time, to avoid drugs with synergistic ototoxic effects and to perform regular audiological monitoring throughout treatment. Audiologic testing facilities are virtually non-existent in many countries in Africa and elsewhere in the developing world. Identifying and only testing those patients at-risk of aminoglycoside induced ototoxicity would lead to more efficient use of limited audiologic facilities. It is also imperative that all maternal relatives, since these are mitochondrial mutations, of mutation-positive individuals should be counselled about their propensity to develop irreversible hearing impairment if they are treated with aminoglycosides.

## Conclusion

In the present study we have developed a cost-effective method using the SNaPshot technique that in a single reaction detects five of the known mutations associated with aminoglycoside-induced hearing loss. This test would facilitate the detection of susceptible individuals prior to the start of their aminoglycoside therapy and so potentially could lower the incidence of this type of deafness.

## Abbreviations

ASO: allele-specific oligonucleotide hybridisation; ddNTPs: dideoxynucleotide triphosphates; MDR-TB: multidrug-resistant tuberculosis; PCR: polymerase chain reaction; RFLP: restriction fragment length polymorphism; SAP: shrimp alkaline phosphatase; WHO: World Health Organisation.

## Competing interests

The authors declare that they have no competing interests.

## Authors' contributions

SB designed the study, directed the genetic analysis and drafted the manuscript. HH, GH and RV performed the molecular genetic studies. HH contributed to the sample collection. TH, HSS and JF participated in the design of the study and helped to draft the manuscript. LvdM performed the statistical analysis and critically reviewed the manuscript. JHG provided some of the mutation positive controls and critically reviewed the manuscript. GdJ conceived of the study, and participated in its design and coordination and helped to draft the manuscript. All authors read and approved the final manuscript.

## Pre-publication history

The pre-publication history for this paper can be accessed here:


